# Optical Aggregation of Gold Nanoparticles for SERS Detection of Proteins and Toxins in Liquid Environment: Towards Ultrasensitive and Selective Detection

**DOI:** 10.3390/ma11030440

**Published:** 2018-03-17

**Authors:** Antonino Foti, Cristiano D’Andrea, Valentina Villari, Norberto Micali, Maria Grazia Donato, Barbara Fazio, Onofrio M. Maragò, Raymond Gillibert, Marc Lamy de la Chapelle, Pietro G. Gucciardi

**Affiliations:** 1 CNR-IPCF, Istituto per i Processi Chimico-Fisici, Viale F. Stagno D’Alcontres 37, I-98168 Messina, Italy; antonino.foti@polytechnique.edu (A.F.); c.dandrea@ifac.cnr.it (C.D.A.); villari@ipcf.cnr.it (V.V.); micali@ipcf.cnr.it (N.M.); donato@ipcf.cnr.it (M.G.D.); fazio@ipcf.cnr.it (B.F.); onofrio.marago@cnr.it (O.M.M.); 2 Dottorato di Ricerca in Fisica, Università di Messina, Viale F. Stagno D’Alcontres 31, I-98166 Messina, Italy; 3 Laboratoire CSPBAT, Université de Paris 13, Sorbonne Paris Cité, CNRS, 74 Rue Marcel-Cachin, F-93017 Bobigny, France; raymond_gillibert@yahoo.fr (R.G.); marc.lamydelachapelle@univ-lemans.fr (M.L.d.l.C.); 4 Institut des Molécules et Matériaux du Mans (IMMM-UMR CNRS 6283), Université du Mans, Avenue Olivier Messiaen, 72085 Le Mans, France

**Keywords:** SERS, biosensor, gold nanoparticles, aptamers, toxins, hemeprotein, optical forces, optical tweezers, optical patterning, colloids

## Abstract

Optical forces are used to aggregate plasmonic nanoparticles and create SERS–active hot spots in liquid. When biomolecules are added to the nanoparticles, high sensitivity SERS detection can be accomplished. Here, we pursue studies on Bovine Serum Albumin (BSA) detection, investigating the BSA–nanorod aggregations in a range from 100 µM to 50 nM by combining light scattering, plasmon resonance and SERS, and correlating the SERS signal with the concentration. Experimental data are fitted with a simple model describing the optical aggregation process. We show that BSA–nanorod complexes can be optically printed on non-functionalized glass surfaces, designing custom patterns stable with time. Furthermore, we demonstrate that this methodology can be used to detect catalase and hemoglobin, two Raman resonant biomolecules, at concentrations of 10 nM and 1 pM, respectively, i.e., well beyond the limit of detection of BSA. Finally, we show that nanorods functionalized with specific aptamers can be used to capture and detect Ochratoxin A, a fungal toxin found in food commodities and wine. This experiment represents the first step towards the addition of molecular specificity to this novel biosensor strategy.

## 1. Introduction

Surface Enhanced Raman spectroscopy (SERS) [1,2] has proven to be an extraordinary tool for direct (*label-free*) detection of biological entities and biomedical applications [3,4,5]. The huge amplification of the electric field provided by the resonant excitation of localized surface plasmons (LSP) [6,7] can bypass issues related to the low non-resonant Raman scattering cross-section of biomolecules in the visible range (σ_scatt_ ~ 10^−30^ cm^−2^ molecule^−1^ sr^−1^) [8]. The SERS effect can be an efficient transducer among the class of optical biosensors, giving access to the spectroscopic information, useful to get insight on the interactions of biomolecules with their environment and to know their structure and conformation [9,10]. Label-free SERS detection of proteins in liquid environment has the further advantage of keeping proteins in their natural habitat. Creation of SERS–active aggregates in a solution containing biomolecules, without affecting their functionality, is still a challenge [9]. Gold or silver colloidal solutions are the most suitable systems for in-liquid SERS detection, representing a three-dimensional matrix that closely interacts with the analytes in their native form when they are mixed together [11]. Individual nanoparticles (NPs) do not provide enough amplification for SERS detection of biomolecules. NP clusters, conversely, can be extremely effective in accomplishing such a task [3,12]. When the gap between the NPs is below 10 nm, in fact, the SERS enhancement factor (EF) at the interstices of the nanostructures (hot spots) can increase by a factor 10 to 10^4^, thanks to the rise of new gap plasmon resonances [1,13]. Obtaining a controlled aggregation of plasmonic colloids with analytes located in hot-spots is therefore a crucial point for the fabrication of an ultra-low sensitive SERS sensors working in liquid environment. Water in the interstices among the nanoparticles prevents the colloidal system from collapsing, and then causing a strong reduction of the EF [14] due to quantum plasmonic effects [15,16]. This allowed reaching attomolar sensitivity [17,18], demonstrating that “wet” plasmonic aggregates can be more efficient with respect to the dried ones. Simple ways to aggregate colloidal plasmonic nanoparticles involve the addition of external chemical agents, such as salts [19] or pyridine [20]. Such methodologies can provide very strong SERS amplification (~10^6^–10^11^) [1] but also large signal instabilities and lack of reproducibility. Inducing the NP aggregation via addition of acidified sulfate to the solution containing the target protein yields SERS–active colloid–protein complexes in which the biomolecules are located at the NPs hot spots [3]. With this strategy, quantitative detection of non-resonant proteins was achieved at concentrations down to 5 μg/mL on Lysozime. Chemicals can also be added to a protein solution mixed with NPs to create NP–protein–NP structures at the interface of an optical fiber immersed inside the sample pushing the limit of detection down to 0.2 μg/mL [4]. However, an acidic environment (pH 3) is needed and this dramatically alters the natural configuration of the biomolecules. Iodide-modified Ag NPs (Ag IMNPs) [10] aggregate by protein addition, making the detection process more biocompatible. A limit of detection (LOD) of 3 µg/mL was achieved for Lysozime, and 300 µg/mL for Bovine Serum Albumin (BSA). Aggregation of NPs with biocompatible coatings is also a feasible way for SERS detection (LOD ~ 50 nM for cytochrome C) [21].

Photochemical and photophysical effects induced by light can trigger the formation of SERS–active structures for detection of chemical compounds in liquid [22,23,24]. Remote aggregation and SERS sensing can be achieved by using optical fibers [25]. Aggregation triggered by light irradiation can be reversible when it exploits thermophoretic effects [26] or temperature-responsive coil-to-globule transitions [27], induced by plasmonic heating [28]. The interaction between light and metal NPs is also mediated by optical forces [29,30]. They enable contactless manipulation, providing novel routes to achieve efficient SERS in-liquid [31,32], in a controlled and chemicals-free way [32,33], offering new strategies for single molecule detection [34] and in vivo applications [35]. The nature of the light-particle interaction can be switched from the attractive (optical trapping) to the repulsive regime (optical pushing) [36] by changing the intensity or wavelength of the light beam. When the laser field is far-off the particle’s LSPR, optical forces are dominated by the gradient force and can either attract or repel the metal NPs from the high field intensity regions [37,38]. When light is nearly-resonant to the NPs LSPR, conversely, the radiation pressure prevails and metal NPs are pushed along the beam axis [35,39,40].

Svedberg et al. first showed the possibility to create SERS–active dimers by optical manipulation of metal NPs in liquid, showing SERS signals of organic molecules dissolved therein [41]. This experiment paved the way to new strategies for SERS detection in liquid [42,43] and lab-on-chip microfluidic architectures [44,45] via optical manipulation of colloids, with sensitivities that can reach the fM range for simple molecules. We recently applied optically induced aggregation of gold nanorods to the SERS detection of amino acids and proteins (BSA and Lysozime) in liquid (LIQUISOR) with sensitivity down to 100 nM in concentration, showing the potential of this technique in the field of biomolecular sensing [46].

An ideal biosensor besides being highly sensitive must also feature high specificity and capability of quantitative determination of the amount of the target molecule in solutions. Raman is indeed a quantitative technique. Although SERS suffers from signal variability issues due to the heterogeneity of the hot spots distribution and molecular binding, a proper statistical sampling and advanced data analysis [3] can be used to obtain concentration information [47,48,49]. Highly specific SERS detection of analytes in complex environments, such as body fluids, can be implemented by functionalizing the NPs with bioreceptors such as antibodies or aptamers [50,51,52], i.e., grabbing strategies routinely employed in well assessed indirect biosensors such as quartz crystal microbalance (QCM), enzyme-linked immunosorbent assays (ELISA) and surface plasmon resonance (SPR) [53,54,55]. Thiolated aptamers are DNA strands designed to interact with specific kinds of molecules, on one side, and with gold surfaces on the other. Thiolated aptamers have gained much favor in SERS biosensing because they stably link to gold NPs and because their small dimension (20–100 unit bases) permits to keep the target molecules in the hot spot of the NPs, thus taking advantage of the local enhanced field [56,57,58].

In this article, we examine in more depth the LIQUISOR detection of BSA, correlating the SERS signal with concentration and carrying out a dimensional (light scattering) and spectroscopic study (localized plasmon resonance) of the interaction between BSA and CTAB-capped gold nanorods at concentrations form 100 µM to 50 nM. A model is developed describing the optical aggregation process that fits the power law dependence of the SERS signal on the concentration. Optical printing of BSA–nanorod complexes is demonstrated on non-functionalized glass substrates, writing custom patterns. With the aim of extending the application spectrum of the LIQUISOR methodology, we report first results on hemoglobin and catalase, two Raman Resonant hemeproteins, detected in liquid at concentrations of 10 nM and 1 pM, respectively. Finally, we show first results on the use of nanorods functionalized with specific aptamers for the capture and SERS detection, in liquid, of Ochratoxin A (OTA), a fungal toxin occurring in food commodities and wine. These latter experiments represent the first step towards the addition of high molecular specificity to the LIQUISOR methodology.

## 2. Materials and Methods

### 2.1. Gold Nanorods

We used gold nanorods from Nanopartz (35 nm diameter × 90 nm length and 30 nm diameter × 50 nm length), dispersed in deionized (DI) water at a concentration of 0.05 mg/mL; the solution contains <0.1% ascorbic acid and <0.1% Cetyltrimethylammonium bromide (CTAB) surfactant to prevent re-aggregation. The solution pH varied between 3 and 4. The rods had a positive ζ-potential (+40 mV). Major plasmon resonances were at 690 nm (35 × 90) and at 540 nm (30 × 50).

### 2.2. Glass Microcell

We employed microscope glass slides with single cavities (15–18 mm dia., 0.5–0.8 mm depth, Marienfled GmbH), as microcells. They were sealed with standard 170 µm-thick glass coverslips (Forlab). The microcell accommodates up to 75 µL of NRs-biomolecules solution. Smaller amounts (15 µL) were analyzed with custom-built microcells, made with a bi-adhesive spacer 120 µm thick attached on a glass slide and sealed with a glass coverslip. All glasses were washed by immersion in a deionized watery solution (1% *v*/*v*) of HELLMANEX III detergent for 10–15 min, followed by rinsing in DI water to remove the residual detergent. Finally, they were washed with ethanol and dried in air.

### 2.3. Protein Solutions

BSA, Hgb and Cat were purchased from Sigma Aldrich (Darmstadt, Germany) in lyophilized powder state and diluted in Phosphate Buffered Solution (PB, pH 7.2). PB solutions 200 mM were prepared by dissolving Na_2_HPO_4_ (14.94 g) and NaH_2_PO_4_ (5.06 g) in 200 mL of DI water. BSA is diluted in PB (200 mM) at concentrations ranging from 10^−3^ to 10^−8^ M, and then mixed with nanorods (both families) in a 7:1 *v*/*v* volume ratio. Hgb (10^−4^–10^−12^ M) and Cat (10^−5^–10^−8^ M) were diluted in PB 10 mM, and then mixed with the nanorods (30 nm diameter × 50 nm length) in a volume ratio 4:1 *v*/*v*. Solutions were prepared in 1.5 mL Eppendorfs, from which 80 µL aliquots were pipetted into the glass microcells. All solutions were prepared and used at room temperature.

### 2.4. Gold Nanorods Functionalization

Aptamers for OTA are thiolated single strand DNA (ssDNA) and were purchased from Eurogentec (Seraing, Belgium). They were composed of 36 nucleotides (M_w_ ~ 11.5 kDa) and had the following specific sequence: 5′-HS-(CH_2_)_6_-GAT CGG GTG TGG GTG GCG TAA AGG GAG CAT CGG ACA-3′. This sequence was determined by Cruz-Aguado and Penner [59] and was already used by Galarreta et al. for SERS detection of OTA with a limit of detection of 50 nM [57]. NRs were functionalized with the DNA aptamers with the following protocol. The NRs (30 nm diameter × 50 nm length) were diluted in DI water in a volume ratio of 1:4 *v*/*v*. Aptamers were diluted in PB 10 mM down to a concentration of 1 µM and then mixed with the NRs solution in a volume ratio of 1:3 *v*/*v*. NRs were left to incubate with aptamers for 1 h.

### 2.5. Toxin Solutions Preparation

Ochratoxin A was purchased from Sigma Aldrich (Darmstadt, Germany) in powder state and was diluted in PB 10 mM at the final concentration of 1 µM and mixed with the functionalized NRs in a volume ratio of 3:1 *v*/*v*. The system was left to incubate for 2 h at room temperature.

### 2.6. Dynamic Light Scattering

Dynamic light scattering (DLS) [60] measurements were carried out to obtain dimensional information, namely the mean hydrodynamic radius (MHR), of BSA-nanoparticle complexes in liquid solutions. DLS experiments were carried out with a Photon Correlation Spectroscopy setup. A He–Ne laser source (10 mW), polarized orthogonal to the scattering plane, was focused onto the sample and the scattered light was collected at 90° by using a self-beating detection mode. Two photomultipliers were used for light detection, in a pseudo-cross correlation mode at the same scattering angle. For collection of the polarized and depolarized scattered light, a Glan–Thomson analyzer was placed in the scattered beam. During the experiment, each sample was put in a glass cuvette positioned inside a thermostat to perform the experiment in a thermal equilibrium condition.

### 2.7. Extinction Spectroscopy

Extinction spectroscopy was used to study the aggregation properties of the BSA-NR complexes by monitoring their time- and concentration-dependent plasmon resonances. Extinction spectra were acquired using the internal white light source embedded in the XploRA microspectrometer (Horiba Jobin Yvon, Kyoto, Japan) for excitation. Collection of the light transmitted through the microcell containing the NRs-protein solution was performed with a 10× microscope objective (Olympus M-Plan, NA 0.2) [61]. Detection of the transmitted intensity ($I_{T}$) was accomplished with a Peltier cooled CCD (Sincerity). We considered the absorbance profile to assign the plasmon resonance, calculated as $-\log_{10}\left(I_{T}/I_{0}\right)$, where $I_{0}$ is the excitation intensity. For these experiments, NR-BSA solutions were prepared in a 1 mL eppendorf and 80 µL aliquots were pipetted to the glass microcell for analysis at defined time intervals.

### 2.8. Raman Spectroscopy Setups

LIQUISOR was operated indifferently on different confocal Micro-Raman Spectrometers: a LabRam HR800, a XploRA and a XploRA PLUS (Horiba, Kyoto, Japan). SERS experiments on BSA were carried out mainly on the HR800 at 632.8 nm. Optical printing and SERS of Hgb and Cat were carried out mainly on the XploRA PLUS at 638 nm. Experiments on aptamers and OTA were carried out mainly on an XploRA at 660 nm. The HR800 employed a He-Ne laser source (632.8 nm); the beam was focused by means of a 100× microscope objective (Olympus M-Plan, NA = 0.90, WD = 210 μm) on a ~ 600 nm diameter spot. The laser power on the sample was 5 mW, enough to apply a sufficient radiation pressure on the nanorods for process activation. The XploRA and XploRA PLUS setups used laser diode sources at 660 nm and 638 nm, respectively. Optical aggregation was accomplished with long working distance microscope objectives (Olympus LMPlanFl 50×, NA 0.5, WD = 10.6 mm; Olympus LUCPLFLN 60×, NA 0.7, WD = 1.5 mm), using higher laser power (13–18 mW). In all the cases, the SERS signal was collected via the same illumination objective, in backscattering, dispersed by a 600 lines/mm grating and detected through a Peltier-cooled silicon CCD (Synapse and Sincerity by Horiba Jobin Yvon or Andor iDus DU 420, (Belfast, UK). Spectra were acquired with integration times from seconds to tens of seconds.

## 3. Results and Discussion

### 3.1. LIQUISOR Operation

**Figure 1:**
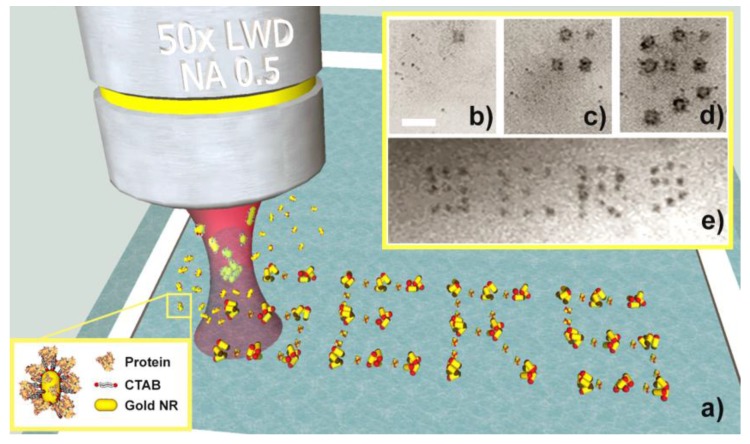
(**a**) Sketch of the LIQUISOR methodology in which a focused laser beam is used to optically aggregate BIO-NRC complexes (inset) dispersed in solution. (**b**–**d**) Optical pictures of BIO-NRC aggregates produced sequentially to print the word “SERS” (**e**) in liquid. Scale bar in (**e**) is 15 µm. Optical printing of each spot requires ca. 3 min.

The working principle of the LIQUISOR [46] is illustrated in Figure 1a. CTAB-coated gold NRs are added to a solution of biomolecules dissolved in PB. Molecular interactions (see Section 3.2) yield the formation of biomolecule-NR complexes (BIO-NRCs) in solution (inset in Figure 1a). This latter is pipetted into a glass microcell and positioned under the Raman micro-spectrometer, where it is irradiated with a laser beam focused by a microscope objective.

**Figure 2:**
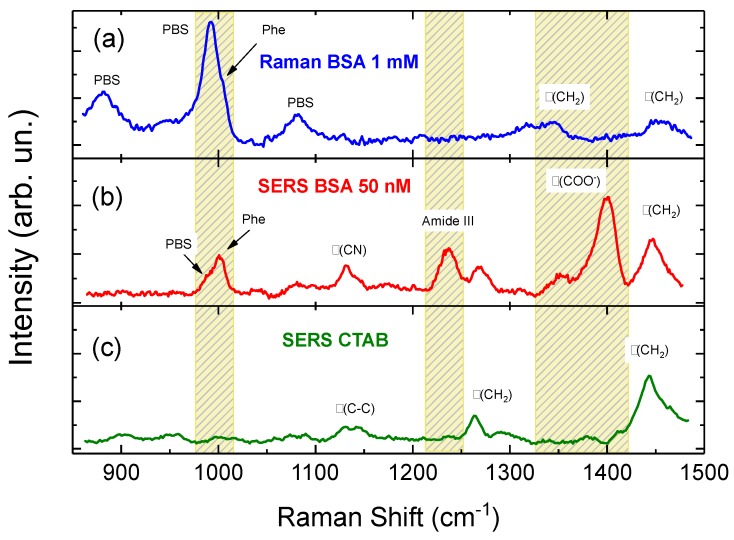
(**a**) Raman spectrum of BSA 1 mM in PB (laser power 6.5 mW, integration 30 s). (**b**) SERS spectrum of BSA 50 nM in PB (laser power 6.5 mW, integration 30 s). (**c**) SERS signal detected on NRs precipitated in absence of protein, attributed to the residual CTAB (laser power 2 mW, integration 120 s).

In our experiments, we use 30 nm × 50 nm NRs featuring a single plasmon resonance at 540 nm (Figure S1, blue line) and 35 nm × 90 nm NRs featuring a short axis resonance at 520 nm (Figure S1, green line) and a more intense long axis resonance at 690 nm. In both NR families, the optomechanical interaction with the laser beams (λ = 633, 638, 660 nm) is dominated by the scattering force, no matter if the field is polarized parallel to the short or to the long axis of the NRs (see calculations in Ref. [46] for the 35 nm × 90 nm NRs and Figure S2 for the 30 nm × 50 nm ones). Positioning the laser spot close to the bottom of the microcell, the BIO-NRCs intercepted by the laser beam are pushed from the focal point towards the microcell surface where they stick and aggregate. It is therefore possible to aggregate the BIO-NRCs in a confined region having dimensions in the 5–10 µm range (black spot in Figure 1b) on time scales ranging from few to some tens of minutes. By displacing the laser spot with a micro- or nano-positioning stage, it is possible to optically print BIO-NRCs on glass, in a sequential way (Figure 1c–e), and without any prior surface functionalization. Virtually any pattern can be printed, provided the spacing among the spots is larger than the average spot size. Both NR families have been used for optical printing. Due to the rod–rod near-field interaction, the aggregates feature very strong field enhancement effects, yielding an intense SERS emission from the biomolecules located inside the hot spot regions. In Figure 2, we compare (Figure 2a) a conventional Raman spectrum of BSA at 1 mM (the minimum detectable concentration in PB), with the SERS signal of BSA in PB at 50 nM, i.e., a concentration 20,000 times smaller, acquired by the LIQUISOR technique (Figure 2b). The SERS signal shows strong contributions in the regions of the Phe ring breathing (1004 cm^−1^), to the amide III (1237 cm^−1^) and to the COO^−^ vibration (see [46] for more details on the modes attribution). Notably, in those regions, the CTAB signal detected from NRs precipitated in PB in absence of the protein (Figure 2c) does not provide any contribution, excluding signal cross-talks. The positioning of the laser focus close to the bottom of the microcell is critical. No aggregation or SERS signal is observed when the laser spot is focused into the solution.

### 3.2. NRs-Biomolecules Interactions in Buffered Solutions

Upon mixing, the biomolecules bind to the gold NRs [62]. Due to the interplay between the electrostatic interaction of molecules surrounding the NRs with the positively charged CTAB bilayer and the destabilization of the CTAB bilayer induced by the PB at physiological pH, amino acid residues of the protein get in close contact with NRs gold surface [63,64,65,66]. This yields the formation of biomolecule–NR complexes [65,67] in which individual NRs are stabilized by the protein layer in the solution. The formation of the BIO-NRCs was studied on BSA, which is a protein rich of sulfur spread over 17 disulfides (S–S), 5 methionine (S–CH_3_) and one free thiol (a cysteine residue) [65]. Disulfide bridges contribute to a stable protein tertiary structure as they give solidity the α-helix bundles. When they interact with gold nanoparticles, the high sulfur affinity with gold causes the disruption of the S–S bridges and the fast creation of the Au–S coordination [68]. As a result, the protein forms a corona all-around the gold nanostructure changing its tertiary structure [65]. Stable colloidal suspensions are obtained if the protein quantity is sufficient to recover the surface and prevent attractive interaction causing precipitation [65]. This is also the case of NRs covered with CTAB. When we mix BSA (10^−4^ M) in PB (200 mM in water), the high ionic concentration and the pH value of 7.2 causes the destabilization of the CTAB bilayer surrounding the NRs [63]. The presence of BSA and its fast interaction with the gold surface (they form a stable bound in less than 1 µs [65]) ensures a new protein capping layer that can stabilize the suspension.

**Figure 3:**
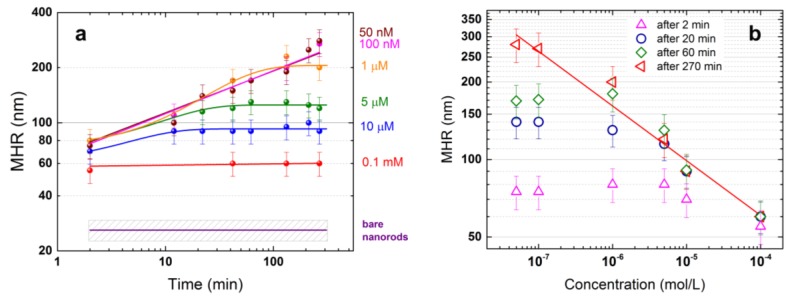
(**a**) Mean hydrodynamic radius (MHR) of BIO-NRCs as a function of time at BSA concentrations of 0.1 mM (red dots), 10 µM (blue dots), 5 µM (green dots), 1 µM (orange dots), 100 nM (pink dots) and 50 nM (brown dots). The solid colored lines are fits of the experimental data using a model $r\left(t\right)=r_{0}+A\left(1-e^{-t/t_{0}}\right)$. Best fit parameters are: $r_{0}$ = 70 ± 20 nm, A = 140 ± 20 nm, $t_{0}$ = 45 ± 20 min (orange line, 1 µM); $r_{0}$ = 72 ± 7 nm, A = 53 ± 7 nm, $t_{0}$ = 11 ± 3 min (green line, 5 µM); $r_{0}$ = 60 ± 10 nm, A = 35 ± 10 nm, $t_{0}$ = 5 ± 3 min (blue line, 10 µM). The purple line represents the MHR measured on the CTAB-coated pristine NRs. Data at 100 nM and 50 nM are fitted with a power law $r\left(t\right)\sim t^{b}$ with b=0.23±0.01 for both concentrations (pink and brown lines). (**b**) MHR as a function of protein concentration after 2 min (pink triangles), 20 min (blue circles), 60 min (green diamonds) and 270 min (red triangles) from the mixing. (red line) Power law fit of the data. Best fit exponent −0.21 ± 0.02.

To get insight on the concentration dependence of the SERS signal in the LIQUISOR experiments, we have first studied the BSA-NR complexes’ size as a function of the protein amount. We use DLS to measure the mean hydrodynamic radius of the complexes in the 0.1 mM–50 nM concentration range. In this range, BSA cannot be detected by conventional Raman, whereas strong SERS is found using the LIQUISOR approach [46]. BSA molecules in PB at 0.1 mM have a mean hydrodynamic radius (MHR) *r*_0_ ~ 6 nm at room temperature [46]. The scattering is almost totally polarized indicating that BSA is in the folded conformation. Upon addition of gold NRs (30 × 50), the MHR increases to $r_{0}=\left(60\pm 10\right)$ nm (Figure 3a, red symbols). This value is ca. 2.5 times the MHR of the pristine CTAB-coated NRs (Figure 3a, purple line). The size is constant over our entire observation period (270 min), indicating that the BIO-NRCs, after a very fast uptake of BSA from the solution, are stabilized. A different behavior is observed at lower BSA concentrations. At 10 µM, 5 µM and 1 µM, the size of the complexes grows with time (blue, green, orange symbols in Figure 3a), reaching a steady state after some minutes. Fitting the data with a simple growth model $r\left(t\right)=r_{0}+A\left(1-e^{-t/t_{0}}\right)$, with *r*_0_ the initial MHR, *A* the size increase and *t*_0_ the process timescale, we find that the stabilization of the BIO-NRCs dimensions takes few minutes at 10 µM ($t_{0}=5\pm 3$ min) and ca. 10 min ($t_{0}=11\pm 3$ min) at 5 µM. At 1 µM the process is slower ($t_{0}=45\pm 20$ min). In addition, larger complexes are obtained at 1 µM: the MHR (200 nm) almost doubles with respect to the complexes obtained at 10 µM (90 nm) and triples with respect to ones at 100 µM (60 nm). At the lowest concentrations (100 and 50 nM, purple and brown symbols in Figure 3a), the MHR of the BIO-NRCs keeps increasing. A stable configuration is never reached within our maximum observation period (270 min). In this ranges, data are well fitted with a power law model $r\left(t\right)\sim t^{b}$, with b=0.23±0.01 for both concentrations (pink and brown lines). In Figure 3b, we plot the size of the complexes vs. BSA concentration at four different time intervals, i.e., soon after mixing (2 min, pink triangles), in a time scale typical of the LIQUISOR experiments (20–60 min, blue circles and green triangles) and in a timescale (270 min, red triangles) larger that the longest LIQUISOR acquisitions (typically 60–120 min). Two minutes after mixing, the complexes have MHR between 60 and 75 nm (error is between 5 and 10 nm). After 20 min, the size is unchanged (60 ± 10 nm) at 100 µM, while it increases to values between 90 ± 10 nm (10 µM) and 140 ± 20 nm (1 µM and lower concentrations).

After 270 min, the complexes formed at concentrations below 1 µM have further increased their dimension to 200 ± 30 nm (1 µM), 260 ± 30 nm (100 nM) and 270 ± 30 nm (50 nM), whereas at higher concentrations (5 µM, 10 µM and 100 µM) no appreciable size increase is observed. Fitting the data with a power law $r\left(c\right)\sim c^{b}$ (red line in Figure 3b), we find an exponent value of −0.21 ± 0.02. This power law also holds on time scales between 20 and 60 min at concentrations of 1–100 µM. Notably, we find that the scattering intensity at 90° is constant with time for concentrations down to 5 µM, while a steep decrease is observed at lower concentrations (data not shown). The scattering decrease at 90° is justified, indeed, by the precipitation of the nanoparticles, that would lead to a smaller number of scattering events per unit time. In principle, however, we cannot exclude that a change of the particle’s form factor among the causes of the scattering intensity decrease at 90°, although LSPR measurements (vide infra) support the precipitation hypothesis.

**Figure 4:**
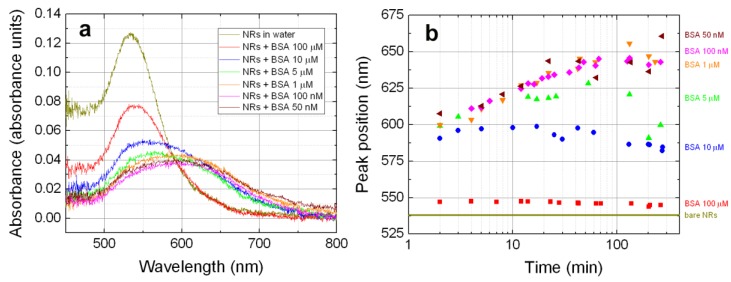
(**a**) Plasmon resonance of the CTAB-coated NRs diluted in water (gold line) and after few minutes from mixing with BSA in PB at different concentrations (other colored lines). (**b**) Position of the red-shifted component of the plasmon resonance of the BSA-NR complexes as a function of time for different BSA concentrations. In all fits, the position of the 540 nm peak (not shown) is constant within ±3%.

Extinction spectroscopy becomes crucial at this stage to understand the nature of the growing objects in solution. To this aim, we have studied the plasmon resonance of the complexes formed on time scales and protein concentrations relevant for the LIQUISOR operation (from few minutes to some hours and from 100 µM to 50 nM). In Figure 4a (dark yellow line), we show the resonance profile of the NRs diluted in water compared with the ones obtained after mixing with BSA in PB (colored lines). Within the first 3 min from the mixing, the resonance red shifts and broadens. At 100 µM (red line), the shift is limited to some nm and the resonance profile is still well fitted by a single peak (Figure S3a, green dots and blue curve). This suggests that many individual NRs populate the solution. Considering that the dimension observed at this concentration is ~60 nm, we probably have the formation of a protein multilayer around the NRs surface. Decreasing the concentration, the resonance remarkably red shifts and broadens (Figure 4a blue, green, orange, magenta, brown lines), likely due to coupling effects among NRs that form aggregates very rapidly (few minutes or less) [46,69]. We phenomenologically describe the resonance profiles of such a system with a two-component Gaussian, one peaked at ~540 nm and a second one red-shifted and broadened. Such a model fits the data well at all concentrations (see Figure S3b). Measurements as a function of time show that the LSPR properties of the complexes are strongly concentration-dependent. In Figure 4b, we plot the position of the second, red shifted peak as a function of time, finding a behavior that closely resembles the one observed in the DLS measurements (Figure 3a). At 100 µM (red squares), the resonance wavelength is almost constant, suggesting that the protein quantity is enough to stabilize the NRs at the single nanoparticle level. At 10 µM (blue circles), after an initial slight red-shift of the resonance, we observe a steady state and a final blue shift (on time scales exceeding 100 min). This behavior is more pronounced at 5 µM (green triangles), with an initial red-shift of the resonance and a final blue shift. NR aggregates between 90 and 150 nm are present in the solution at these concentrations, stabilized by an external protein layer. Very likely, a smaller number of NRs, not fully covered by the protein, form unstable aggregates that precipitate at times larger than 100 min, leaving in the solution only smaller objects. This can justify both the fictitious blue shift effect observed in the LSPR profiles and the slight MHR decrease (although within the error bar) observed at 5 and 10 µM (Figure 3a).

At lower concentrations (1 µM to 50 nM), the red-shift is continuous with time (orange, magenta and brown symbols) and rather independent from the specific concentration. Some saturation is observed only on time scales exceeding 100 min. At these concentrations, the quantity of protein is insufficient to stabilize the complexes. Larger and larger NR aggregates (MHR > 200 nm) are formed in the solution, which precipitate on longer time scales. Similar to what was observed in the scattering intensity at 90°, here, the integrated absorbance is almost constant down to 5 µM and starts decreasing with time at lower concentrations, supporting the occurrence of precipitation phenomena. Spectra acquired after one day (data not shown), in fact, show that at 1 µM and below the absorbance is almost zero, confirming that the quasi totality of the NR aggregates is precipitated, whereas stable LSPR fingerprints are found at concentrations of 5 µM and higher.

On the time scales relevant for LIQUISOR operation (5–100 min) we can conclude that at 100 µM the BIO-NRCs solution is mainly composed by individual NRs and by NR aggregates at 10 and 5 µM, both stabilized by a protein corona. At lower concentrations (1 µM down to 50 nM), we have larger and larger NR aggregates with a protein surface coverage insufficient to stabilize them. Notably, the plasmon resonance peak is always close to the laser wavelength, ensuring that the scattering force is always prevalent, and gives rise to optical forces that push the BIO-NRCs along the beam propagation direction at any concentration and time.

Our experiments suggest a picture in which three concurrent processes contribute to the formation of the BIO-NRCs: (i) the destabilizing effect of the PB that causes aggregation of the NRs with a consequent size increase and red-shift of the plasmon resonance; (ii) the surface coverage of the protein that replaces the CTAB and stabilizes the NRs to some steady dimensions and plasmon resonance wavelengths; and (iii) the precipitation of aggregates not fully stabilized by the protein that yields the fictitious blue shift of the LSPR observed on the longest time scales. At high concentration, BSA is enough to cover the surface of the NRs almost immediately, stabilizing the colloids and avoiding any relevant aggregation. As we decrease the concentration in the micromolar range, the probability for the analyte to bind to the NRs surface is smaller [70], and then the time necessary to totally cover and stabilize the BIO-NRCs increases. Meanwhile, the PB keeps on destabilizing the CTAB micellar capping, leading to the aggregation of NRs before the formation of a stabilizing protein corona. BSA, having several accessible sulfurs on its surface, can also act as a bridge between different nanorods also fostering the aggregates formation. Aggregates that are not fully stabilized by the protein form larger structures that do precipitate. At lowest concentrations (1 µM–50 nM), the quantity of protein is insufficient to cover the BIO-NRCs surface, provoking a continuous size increase of the aggregates, a process that ends up with precipitation of the structures. Aggregation of gold NRs upon addition of µM quantities of BSA was observed [69,71] and attributed to an unfolding of the BSA at the NRs surface followed by hydrophobic patch assembly [71]. An estimation of the number of proteins attached to the NRs can be carried out applying the “difference-volume” method reported in Refs. [46,72] to our DLS data. At 100 µM BSA concentration, where complexes consisting of individual NRs are formed, we assume that the MHR increase measured upon addition of BSA is entirely due to protein uptake. To estimate the average number of proteins attached to each NR, we divide the additional volume $\Delta V=V_{\mathrm{NR}-\mathrm{BSA}}-V_{\mathrm{NR}}$ measured by DLS, where $V_{\mathrm{NR}-\mathrm{BSA}}$ is the volume of the NR-BSA complex and $V_{\mathrm{NR}}$ is the volume of the NR, by the BSA volume $V_{\mathrm{BSA}}=4\pi /3\;R_{\mathrm{BSA}}^{3}$, assuming that the radius $R_{\mathrm{BSA}}$ equals the MHR measured by DLS. Circa 900 ± 150 BSA molecules are estimated to surround the NRs surface, enough to cover 100% of the surface, corresponding to ca. 6 layers. At 10 and 5 µM BSA concentrations, we have stable NR aggregates. The volume of the complexes at steady state is, respectively, three and ten times larger than those formed at 100 µM. In these conditions it is not straightforward to disentangle the quantity of protein from the quantity of NRs that contribute to the total volume of the complex.

### 3.3. SERS Intensity vs. Concentration

**Figure 5:**
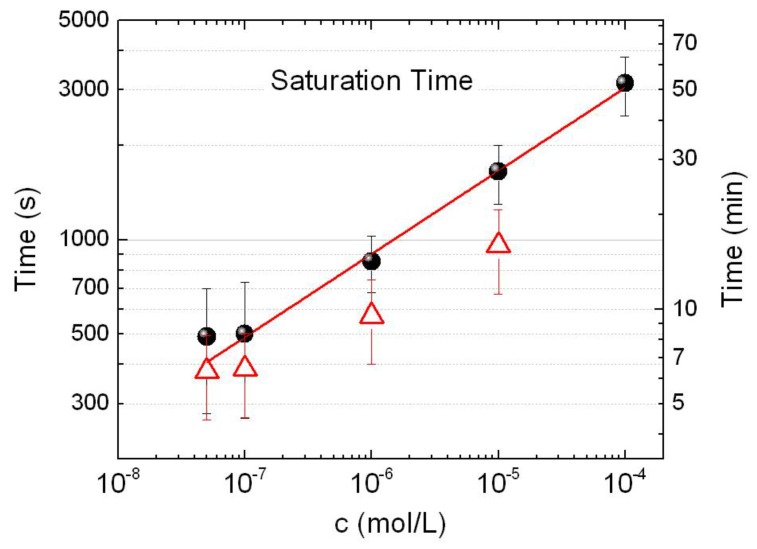
Saturation time (black dots) plotted against BSA concentration. Each point is the average of the saturation times for at least three different aggregates. Red line is power law fit with exponent 0.26 ± 0.01 (best fit parameter). Red symbols are estimated values for *T_sat_* from the hydrodynamic model in Supplementary Note 1.

In Ref. [46], we have already shown that, at 100 µM BSA in PB, the rise of the SERS signal occurs through several steps: (i) the onset of the process in which first aggregates form on the cell walls and yield some detectable SERS signals (this phase takes from few tens of seconds to some minutes from the beginning of the irradiation); (ii) a stabilization phase which produces a stronger, well distinct, SERS signal (taking place on time scales of few tens of seconds); (iii) a repeated increase of the aggregate size (minutes to tens of minutes) due to the capture of further BIO-NRCs, with a continuous growth of the SERS signal; and (iv) a saturation (several tens of minutes, up to one hundred minutes) in which the BIO-NRCs have totally filled up the laser focus and the SERS signal becomes constant. Here, we analyze the SERS dynamics at decreasing BSA concentrations. We acquire spectra at regular intervals under laser irradiation and focus the attention on the time dependence of the Phe peak intensity at 1004 cm^−1^ [46]. To assess the reproducibility of the SERS signal, for each concentration, we analyze at least four different aggregates. A first analysis is carried out on the saturation time, i.e., the time elapsed from the activation of the process (first detectable signal) to the full saturation of the signal. In Figure 5 (black circles), we observe that decreasing the protein concentration, *c*, the SERS signal takes less time to saturate, following a power law dependence with exponent 0.26 ± 0.01 in the range from 0.1 to 100 µM (red line in Figure 5). This behavior can be understood based on the DLS and LSPR measurements, as discussed below, and is confirmed by the simple hydrodynamic model described in Supplementary Note 1. We remark, here, that the saturation time is different from the time interval needed to trigger the aggregation process, which, instead, generally increases when decreasing the concentration.

**Figure 6:**
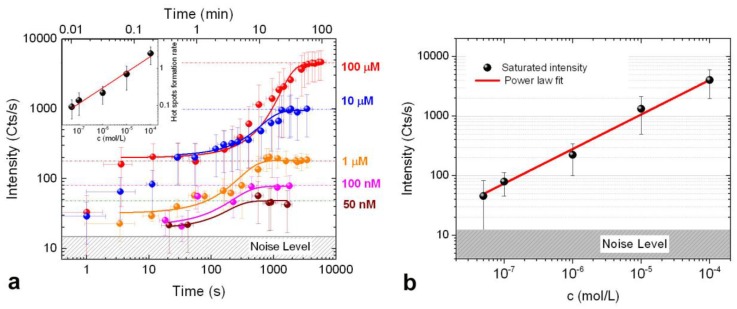
(**a**) Time evolution of the SERS intensity at different BSA concentrations (colored dots). The time *T*_0_ = 0 is set at the onset of the process, when the first weak SERS signal is detected (real-time observation). Starting from *T*_0_ the signal is recorded at intervals corresponding to the integration time used in each spectrum. Solid lines are fits of data using a Boltzmann growth kinetics equation. (inset) Plot of the maximum signal growth rate vs BSA concentration. The solid red line is a power law fit with an exponent 0.40 ± 0.04 as best fit parameter. (**b**) Plot of the SERS intensity at saturation vs. BSA concentration (symbols). The red line is a power law fit with exponent 0.60 ± 0.04.

Figure 6a displays the time evolution of the SERS signal for each concentration as a function of the irradiation time, starting from the activation of the process. It highlights how the LIQUISOR dynamics is qualitatively similar for each concentration, with an onset, a phase of signal increase and a final saturation. The SERS signal increases in a step-like fashion at the onset, while during the intermediate phase it shows a continuous increment. The signal growth is slow at the beginning, it becomes faster after some hundreds of seconds (when the growth rate reaches its maximum) and then slows down again, when approaching saturation. Intensity fluctuations from one aggregate to another, caused by non-uniformity of the enhancement, are encoded in the error bars. Although large, a clear discrimination among the different concentration is obtained at saturation. The SERS intensity curves in the intermediate phase of the process are well fitted with a Boltzmann growth kinetics equation [34] (solid lines in Figure 6a), given by:

(1) $$I=\frac{I_{0}-I_{\mathrm{sat}}}{1+e^{\left(t-t_{0}\right)/dt}}+I_{\mathrm{sat}},$$

where $I_{0},\;I_{\mathrm{sat}}$ define the initial and final (saturation) value of the SERS intensity *I*. $t_{0}$ is the time at which *I* reaches half of its saturation value and also represents the time at which the signal growth rate (curve slope) is maximum. The quantity $v=\left(I_{\mathrm{sat}}-I_{0}\right)/4dt$ measures the curve slope at $t_{0}$ and, therefore, corresponds to the maximum velocity of the SERS signal growth. From the physical point of view, *v* can be considered as the speed at which the hot spots are created, which is related to the nanoparticle aggregation rate in the laser spot. From the fitting of the curves, we retrieve the values of *v* and *I*_sat_ as a function of the concentration, plotted, respectively, in the inset of Figure 6a (black symbols) and in Figure 6b (black symbols). Both *v* and *I*_sat_ increase as a function of the protein concentration with a power law dependence featuring exponents 0.40 ± 0.04 and 0.60 ± 0.04, respectively (red lines in inset of Figure 6a and Figure 6b are the result of the fits). The same trend (Figure S4 and Table S1) is observed analyzing the intensity of other distinctive BSA spectral features, i.e., the Amide III (1240, 1275 cm^−1^) and Phe+Tyr modes (1585–1620 cm^−1^), supporting the idea that the BSA configuration in the interaction with the NRs surface does not change with the protein concentration.

The experimental results can be interpreted, at least qualitatively, making some simple considerations on the origin of the SERS signal. Indeed, the SERS effect is triggered by the field enhancement provided by hot spots in plasmonic nanoparticles. Optical aggregation of 2D layered materials in presence of BSA at 100 µM, in fact, does not provide any Raman fingerprint of the protein [73], indicating that the mere accumulation of protein molecules in the focal spot, brought-in by the nanostructures, is not is not sufficient to provide a detectable signal. The SERS intensity measured at time *t* will scale with the number of hot spots present in the focal spot volume. Saturation of the SERS signal is achieved when the laser focus volume is filled with BIO-NRCs. From DLS, we know that at lower BSA concentrations the size of the BIO-NRCs in solution is larger. Therefore, fewer BIO-NRCs will be sufficient to saturate the laser focal volume. If we assume that the number of hot spots created in the focal region is proportional to the number of BIO-NRCs optically pushed independently from their size, the lower SERS intensity at saturation observed when decreasing the BSA concentration will be a direct consequence of the reduced number of hot spots. This hypothesis can also justify the fact that the saturation condition is reached more rapidly at lower concentrations (Figure 5), since filling the laser spot volume with a smaller number of larger BIO-NRCs will require less time. We can derive a simple physical model out of this picture. We call *I*_sat_ the saturated SERS signal, $N_{\mathrm{NP}}^{\mathrm{sat}}$ the number of nanoparticles present at saturation in the focal spot volume *V*_las_, *R*_H_ the MHR of the nanoparticles, *V*_NP_ the average particle’s volume and, finally, *N*_HS_ the number of hot spots. From the considerations made above, we assume that $I_{\mathrm{sat}}\propto N_{HS}$ and $N_{HS}\propto N_{\mathrm{NP}}^{\mathrm{sat}}$. From simple geometrical considerations, the number of structures in the focal volume is given by the volume ratio $N_{\mathrm{NP}}^{\mathrm{sat}}\propto V_{\mathrm{las}}/V_{\mathrm{NP}}$. The volume of a nanostructure can be calculated from its hydrodynamic radius as $V_{NP}\propto R_{H}^{3}$. Therefore, we expect

(2) $$I_{\mathrm{sat}}\propto N_{\mathrm{NP}}^{\mathrm{sat}}\propto R_{H}^{-3}$$

Assuming the asymptotic concentration dependence of the MHR, $R_{H}\propto c^{-0.2}$, found by DLS (Figure 3b) we obtain

(3) $$I_{\mathrm{sat}}\propto c^{0.6}$$

in agreement with the experimental data (Figure 6b). Notably, such a steep dependence permits distinguishing BSA at different concentrations in a broad range, from 50 nM to 100 µM, despite the large signal fluctuations observed (up to 40%). Moreover, this monotonic dependence of the SERS signal from the concentration opens new possibilities to perform quantitative detection with the LIQUISOR methodology.

Concerning the time needed to saturate the SERS intensity, we expect that *T*_sat_ is proportional to the number of hot spots present at saturation in the focal area, *N*_HS_, and inversely proportional to the velocity at which the hot spots are created, *v*_HS_. Again, if we assume $N_{HS}\propto N_{\mathrm{NP}}^{\mathrm{sat}}\propto c^{0.6}$ and take for *v*_HS_ the velocity of the SERS signal growth determined experimentally (see inset of Figure 6a), $v\propto c^{0.4}$, we find

(4) $$T_{\mathrm{sat}}\propto N_{\mathrm{NP}}^{\mathrm{sat}}/v\propto c^{0.2}$$

This trend is close to what found by fitting the experimental data, $T_{\mathrm{sat}}\propto c^{0.26}$ (Figure 5, red line). A more detailed description of this aspect is developed in Supplementary Note 1.

A final comment regards the way in which the hot spots are formed in the LIQUISOR and how this affects the limit of detection. With BSA at 100 µM, the stability of the dispersion, the single-particle resonance profile of the BIO-NRCs in solution and the LSPR broadening observed upon aggregation [46], led us to conclude that the hot spots are, indeed, induced optically, with an interplay between pushing and thermal effects. At lower concentrations the solutions are populated with clusters of NRs spontaneously formed with proteins distributed on their surface. We expect that some of these are “hot clusters” i.e., SERS–active structures with protein molecules embedded in the hot spots [74]. To observe a strong SERS signal, however, we need to induce some optical aggregation. Focusing the laser in the liquid solution, e.g., at the center of the microcell, in fact, does not produce any detectable signal, at any concentration, suggesting that either the number of “hot clusters” in the solution is not relevant or that their SERS enhancement is not large enough. Furthermore, SERS spectra acquired on micron-sized structures spontaneously formed by precipitation after few hours from the mixing of NRs with BSA at 100 nM show no signal in most of the cases. Sometimes a SERS activity is observed, comparable to the one of early stage aggregates created by LIQUISOR, but the signal does not substantially increase with time. At this stage, we do not have a final answer to the question whether the SERS signal at the lowest concentrations originates from an optical accumulation of “hot clusters” or if it is the consequence of new, more efficient hot spots created by the combination of optical pushing and thermally-induced effects in the laser focus. The measurements reported above, however, show unambiguously that aggregates produced optically are much more “SERS efficient” than those precipitated spontaneously, suggesting that the signal observed in the LIQUISOR is not a mere sum of the contribution of BSA-NR aggregates spontaneously formed in the solutions. At concentrations of 10 nM we do not observe any SERS signal on the usual observation time scales, indicating that aggregation and precipitation takes place on much faster time scales than the protein uptake by the NRs. Under these experimental conditions, the LOD is therefore estimated between 10 and 50 nM.

### 3.4. LIQUISOR Detection of Hemeproteins

Hemeproteins are characterized by the presence of the heme group, in which an iron atom is coordinated to four pyrrole nitrogens of protoporphyrin IX and to an imidazole nitrogen of a histidine residue from the globin side of the porphyrin [75]. This group allows the protein to accomplish several important tasks in biological systems, including oxygen transport, electron transfer as well as biological defense and regulation of oxidation mechanisms.

#### 3.4.1. Hemoglobin

Hemoglobin (Hgb) is a globular metallo-protein composed of two *α* and two *β* subunits non-covalently bound. Each subunit chain is bound to one heme group. Raman spectroscopy can discriminate the normal conformational structure from pathological ones [76], based on the vibrational information on the heme group which is Raman-resonant under visible excitation [77]. SERS is capable of single molecule detection of Hgb [78]. However, in-liquid environment, SERS detection is more complicated and the limit of detection is ~100 nM [3,10].

**Figure 7:**
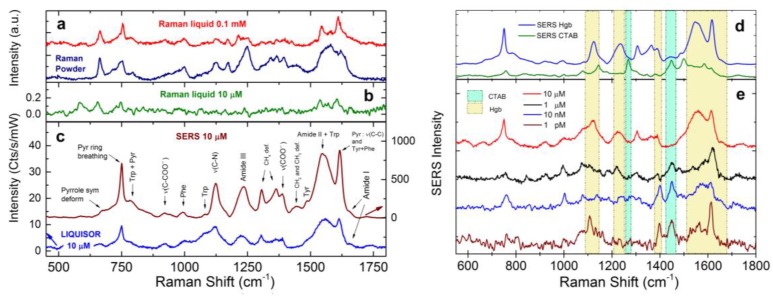
(**a**) Raman spectra of Hgb 0.1 mM in PB (red line—P = 18 mW, T = 60 s, two accumulations) and in powder state (blue line—P = 0.27 mW, T = 60 s, five accumulations); (**b**) Raman spectrum of Hgb 10 µM in PB (P = 18 mW, T = 240 s, two accumulations); (**c**) SERS spectra of Hgb 10 µM acquired with the LIQUISOR methodology (blue line, P = 6.5 mW, T = 1 s) and on a spontaneous aggregate formed after 24 h incubation (brown line—P = 3 mW, T = 1 s); (**d**) reference SERS spectra from aggregates for Hgb (blue line) and CTAB (green line—P = 6.5 mW, T = 1 s, 120 accumulations); and (**e**) SERS spectra of Hgb at decreasing concentrations (P = 6.5 mW, T = 1 s, 50 accumulations). Yellow boxes highlight SERS bands due to the protein while green boxes are due to the CTAB SERS peaks.

To test the potentialities of LIQUISOR on the detection of Hgb, we have carried out experiments by laser irradiation of complexes obtained by mixing Hgb solutions in PB with CTAB-capped gold NR (30 × 50 nm) resonant at 540 nm (Figure 1, blue line). Calculations of the optical forces acting on these NRs at 638 nm with a 50× (NA 0.5) objective highlight (Figure S2) the predominance of the scattering component over the gradient one. The NRs are, therefore, pushed by the laser beam, as confirmed by the experiment. The PB concentration has been reduced to 10 mM because we observed unstable complexes and precipitation phenomena at 200 mM. In these new conditions the CTAB layer is stable on a longer timescale. Hgb, negatively charged, is attracted by the positively charged CTAB bilayer, permitting the formation of complexes also at ultralow concentration, where the uptake of target molecules requires more time. Hemoglobin is diluted at concentrations down to 1 pM and the solutions are incubated with NRs (4:1 *v*/*v*) for 3 h before measurements. Optical aggregation is induced by focusing the laser spot at about 5 µm from the surface of the glass microcell. Laser wavelength is 638 nm. The power is set between 6.5 and 23 mW, depending on the concentration. Once the BIO-NRCs start to aggregate and the first SERS signals is detected, the power is reduced to 3 mW whenever photo-induced damages to the protein are observed (i.e., with the appearance of amorphous carbon bands). Raman spectra of Hgb in both solution (0.1 mM and 10 µM) and powder state have been acquired for reference (Figure 7a,b).

They show the most important vibrational bands of Hgb at 664 cm^−1^ (pyrrole bending), 750 cm^−1^ (pyrrole breathing), 1125 cm^−1^ (CN and CC stretching), 1235 cm^−1^ (amide III), 1393 cm^−1^ (COO^−^ stretching), 1543 cm^−1^ (Amide II + Trp), 1585–1620 cm^−1^ (Phe + Tyr, CC stretching), and 1655 cm^−1^ (Amide I) [74,75,79]. A more detailed list with assignment of the SERS bands of Hgb is reported in Supplementary Table S2. At 10 µM we find the Raman limit of detection of Hgb in PB (Figure 7b, spectrum acquired with 18 mW of laser power and 240 s of integration). At the same concentration, the LIQUISOR methodology permits to detect a clear SERS fingerprint of Hgb after 2–3 min of irradiation, with 1 s integration and 6.5 mW of incident power (Figure 7c, blue line), thanks to the formation of first aggregates of NR complexes at the bottom of the glass microcell. This corresponds to a net signal gain of ~10^2^, considering the modes at in the 1500–1660 cm^−1^. The spectral features well compare with the reference Raman, as well with the SERS signal recorded on aggregates spontaneously precipitated after an incubation time of 24 h (Figure 7c, brown line). In this case, SERS signal gains are ~10^4^ (note that this value is an underestimation of the SERS enhancement factor, whose calculation requires estimation of the number of probed molecules). The SERS of Hgb is definitely different from the signal detected on the precipitated aggregates of NRs without protein (Figure 7d, green line) that we attribute to the CTAB capping agent. Decreasing the concentration of Hgb, we can optically induce SERS aggregates at 1 µM (Figure 7e, black line), 10 nM (blue line), down to 1 pM (brown line). The spectral shape of the amide II (1550 cm^−1^) with the presence of the amide III band (1232 cm^−1^) and C-N stretching modes at 1125 cm^−1^ (highlighted in yellow) give evidence of protein SERS detection even at the lowest concentrations. Another important marker for Hgb detection is the peak at around 1615 cm^−1^, clearly visible also at 1 pM, which is due to the presence of Pyrrole rings of the heme groups included in the protein [77]. Moreover, the SERS signal at 1395 cm^−1^, attributed to the stretching vibration of the COO^−^ group confirms the electrostatic nature of the interaction between proteins and the CTA^+^ heads covering the NRs surface [80]. Note that, as the protein concentration decreases, the bands at 1268 and 1442 cm^−1^ due to CH_2_ wagging and scissoring of the CTAB chains [81] (highlighted by the cyan boxes) appear and get stronger. The residual CTAB signal probably arises from the fact that some surfactant is still attached on the NRs surface. Finally, the different amplification of Hgb bands at the different concentration is likely due to the fact that Hgb does not bind to the gold surface with the same configuration [72], therefore showing different groups to the gold surface. The poor protein quantity at the lowest concentrations prevents a statistical sampling of the whole configurations ensemble, resulting in a different intensity ratio of the protein peaks. Consequently, spectra acquired on different aggregates show non-negligible fluctuations of the peaks intensity, both total and relative. We do not observe, on the other hand, the appearance of new peaks as in single molecule SERS experiments. A signal gain between 10^8^ and 10^9^ is found comparing the SERS intensity at 1 pM of the band at 1617 cm^−1^ due to the C-C stretching of the Pyrrole rings in the heme group with the Raman signal at 10 µM.

#### 3.4.2. Catalase

Catalase (Cat) is an important enzyme for aerobic living organisms that catalyzes the decomposition of hydrogen peroxide to molecular oxygen and water [82]. Cat levels in living organisms can be related to the risk of liver cancer [83] or can be used to monitor the toxic effects of chemical pollutants in marine organisms [84]. SERS permits ultralow detection of Cat in liquid exploiting the chemical aggregation of silver nanoparticles with a LOD of 0.2 nM [3].

**Figure 8:**
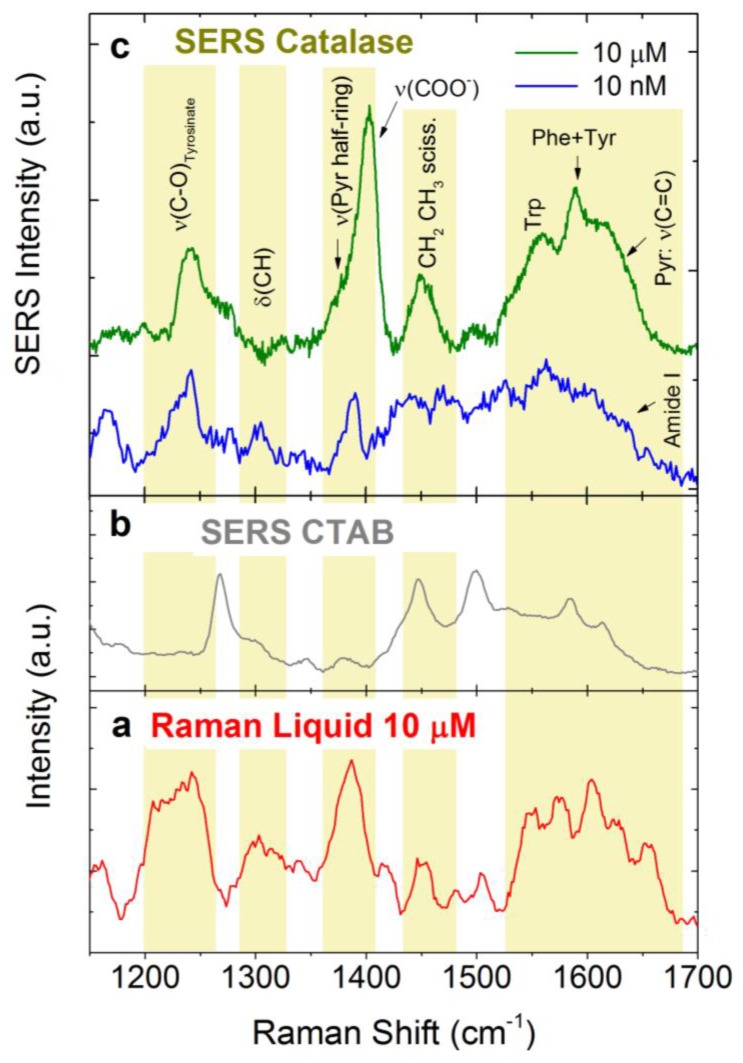
(**a**) Raman spectrum in liquid of Catalase diluted at 10 µM in PB 10 mM (P = 18 mW, T = 240 s, five accumulations); (**b**) SERS spectrum of CTAB (grey line—P = 6.5 mW, T = 1 s, 120 accumulations); and (**c**) SERS spectrum of Catalase acquired with the LIQUISOR methodology at 10 µM (green line, T =1 s P = 3.5 mW, 30 accumulations) and 10 nM (blue line, T =1 s P = 6.5 mW, 30 accumulations). Yellow boxes highlight the vibrational modes due to the protein.

The potentialities of LIQUISOR detection of Cat have been tested diluting the molecule in PB (10 mM). The reference Raman spectrum shown in Figure 8a is acquired on a Cat solution at concentration of 10 µM. The most intense vibrational modes of the protein are the C-O stretching of Tyrosinate-iron group at 1244 cm^−1^, the CH scissoring modes of the vinyl in heme group at 1305 cm^−1^, the pyrrole rings vibrations of the heme group at 1373 cm^−1^ (half ring symmetric stretching), the symmetric stretching of the group COO^−^ at 1390 cm^−1^, the CH_2_ CH_3_ scissoring modes at 1452 cm^−1^ and Amide I band at 1650 cm^−1^. Finally, the band from 1550 to 1620 cm^−1^ is due to the aromatic amino acids (Trp, Phe, and Tyr) and to the pyrrole rings (C=C stretching at 1612–1620 cm^−1^) [3,85]. No Raman signal is detected at 1 µM, suggesting that the LOD of Cat is between 1 and 10 µM. LIQUISOR measurements on Catalase at 10 µM are carried out by mixing the protein solution (in PB 10 mM) with gold NRs (LSPR 540 nm) 4:1 *v*/*v*. The SERS spectrum (Figure 8c, green line) displays an enhancement of the most relevant modes of Cat observed in Raman (highlighted by yellow bands). The same holds for the measurement at 10 nM (blue line in Figure 8c) which shows spectral features distinct from the CTAB signal acquired on precipitated NRs in absence of protein (Figure 8b).

### 3.5. LIQUISOR Detection of Ochratoxin A Mediated by Aptamers

Ochratoxin A is a nephrotic mycotoxin that can be found in different food products, such as cereals, wine, or coffee beans [86,87], representing potential public health risks. The maximum permissible concentration is in the range of 3–5 µg/kg, with a virtual safe dose for humans of less than 0.2 ng/kg per day for renal cancer risk [88]. Aptamer-based SERS sensors have been demonstrated for OTA identification down to 0.1 nM, employing Raman-active reporters [89] or at the concentration of 10 pM with the help of no labels [90], in dry conditions. Implementing the LIQUISOR methodology with the high selectivity of DNA aptamers can open interesting routes for ultra-low-sensitivity and specific SERS detection in liquid environment. Here, we carry out proof-of-concept experiments to show that aptamer-functionalized NRs can be used in the LIQUISOR to detect OTA at micromolar concentration, i.e., below the Raman LOD. Control experiments confirm the crucial role of the aptamer in the capture of the OTA.

The strategy we follow is based on the following steps:

1. Functionalization of the gold NRs with bioreceptors through the substitution of the CTAB layer with thiolated aptamers. This is obtained during incubation of CTAB-coated NRs with aptamers in a PB solution (10 mM). PB destabilizes the CTAB, fostering the interaction between the NRs and the thiol groups of the aptamers.
2. Incubation of the functionalized NRs with the target molecules. The bioreceptors on the NRs surface will selectively capture the target proteins in the solution, creating BIO-NRCs surrounded by a double layer including the aptamers and the captured protein.
3. Normal operation of the LIQUISOR procedure (λ = 660 nm, Objective 60×, NA 0.7). Laser irradiation will produce aggregates in which the target molecules located at the hot spots will experience an enhanced Raman scattering. This latter signal shows up and is compared to the SERS contribution from the aptamers.

**Figure 9:**
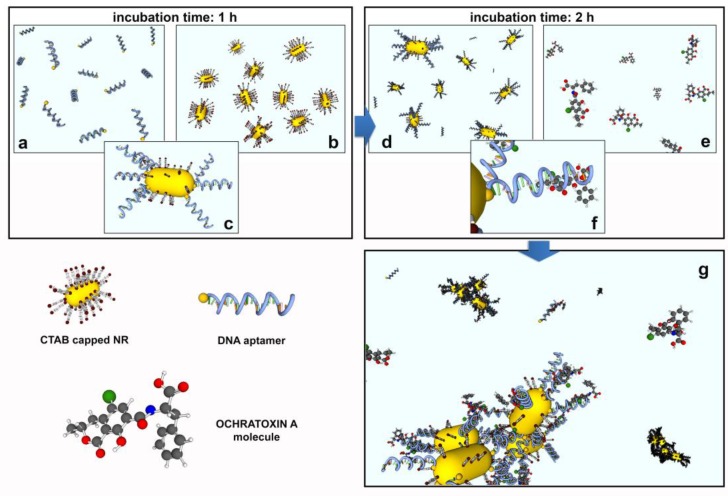
Functionalization process: (**a**) ssDNA aptamer solution is mixed with (**b**) gold NRs in the volume proportion 1:3. After the system has incubated for 1 h, the aptamer functionalized NRs (**c**,**d**) are mixed with the OTA molecules (**e**) in the volume proportion 3:1. The system is left to incubate for about 2 h to allow the interaction between DNA aptamers and molecules (**f**) for the formation of the NR complexes (**g**).

Due to the high affinity between the thiol group of the ssDNA aptamer and the gold surface of NRs, the functionalization process takes advantage of the “one-pot” ligand exchange mechanisms [91] that enables the substitution of the stabilizing molecules (CTAB) with thiolated DNA aptamers (Figure 9c), which is more effective in correspondence of high curvature areas of NRs surface, where the CTAB bilayer has a lower density and ligand exchange processes are energetically more favorable with respect to the side zone of the NRs [90,92].

**Figure 10:**
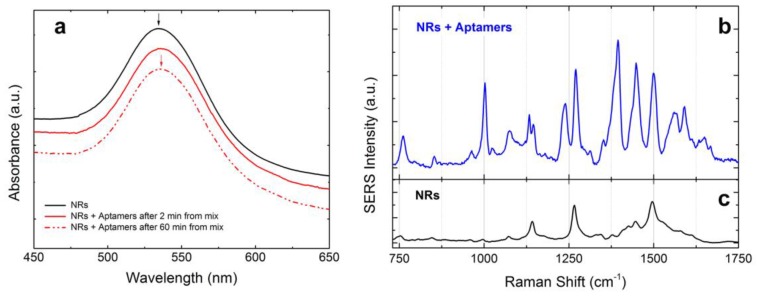
(**a**) Extinction spectra of the CTAB-coated gold NRs (black line) compared to ones functionalized with the aptamers after 2 min of incubation (red solid line) and after 60 min of incubation (red dashed line). The functionalization induces a red-shift of 2 nm that does not change with time. Spectra are offset for clarity. (**b**) SERS signal from the NRs + aptamer complexes optically aggregated at the bottom of the glass microcell (P = 5 mW, T = 1 s). The signal results averaged over 100 consecutive spectra. (**c**) SERS signal (reference) acquired from the CTAB-coated NRs precipitated at the bottom of the glass microcell (P = 1.8 mW, T =1 s) in absence of aptamers.

The functionalization of gold CTAB-coated NRs with aptamers has been monitored by extinction spectroscopy. Interaction between NRs and aptamers yields a 2 nm red-shift of the LSPR peak, after 2-min incubation (Figure 10a, black and solid red lines). The shift and the extinction intensity are constant (dashed red line is the LSPR profile after 1 h incubation), suggesting that the aptamer uptake is very rapid and that the solution is stable with no aggregation phenomena taking place. To verify the success of the functionalization process, we compare the NRs signal with the aptamers SERS coming from optically induced aggregates made with functionalized NRs (Figure 10b). The aptamers signal is consistent with the one reported in Ref. [57], and confirms a predominance of the vibrational modes due to guanine [57], and spectrally different from the background CTAB contribution (Figure 10c).

**Figure 11:**
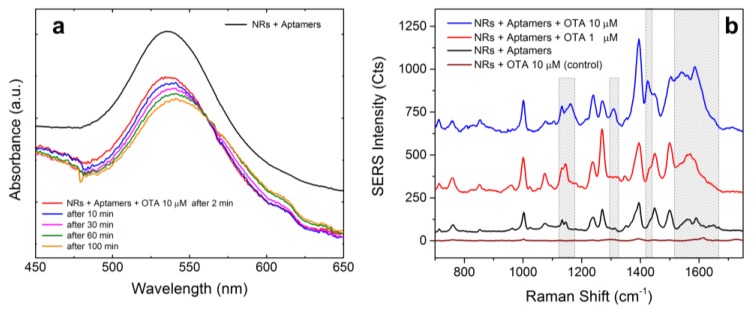
(**a**) Extinction spectra of aptamers-functionalized NRs after 60 min (black line). Addition of OTA (10 µM) causes a progressive shift and broadening of the NRs LSPR (colored lines), due to the interaction between aptamers and OTA molecules. (**b**) LIQUISOR SERS spectrum of aptamers for OTA (black line—P = 5 mW, T = 1 s) compared with the SERS spectrum observed from induced aggregates formed by functionalized NRs mixed with OTA 10 µM (blue line—P = 5 mW, T = 1 s) and 1 µM (red line—P = 5 mW, T = 1 s). Brown line is the spectrum coming out from aggregates spontaneously deposited on the bottom of the glass microcell after mixing NRs and OTA 10 µM without DNA aptamers (P = 18 mW, T = 1 s—in the figure, the spectrum is normalized to P = 5 mW for a better comparison with the other spectra). Dashed boxes highlight the spectral regions where the most evident differences can be observed. All the SERS spectra are averaged over at least 100 consecutive spectra. Data are offset for clarity.

The binding of the functionalized NRs with OTA (10 µM) has been investigated by extinction spectroscopy as a function of the incubation time. As shown in Figure 11a (colored lines), binding with OTA yields a progressive red-shift of the LSPR peak associated with a slight broadening with respect to the spectrum of the NRs + aptamers (black line). The intensity decrease of the peak at 537 nm is associated with a spectral feature at higher wavelengths growing with time, probably indicating the occurrence of some form of aggregation on time scales of 1 h (orange and green line).

LIQUISOR detection of OTA has been proved using functionalized NRs incubated for 1 h with Ochratoxin at concentration of 10 and 1 µM. Optical aggregation and SERS emission is observed with 5 mW laser power after few minutes of laser irradiation. SERS spectra have been acquired with integration times ranging from 1 s to 10 s. The averaged spectra for Ochratoxin 10 µM and 1 µM are displayed in Figure 11b (blue and red lines). The dashed boxes highlight the zones in which the spectra differ from the SERS of the simple aptamer. Main differences are observed from 1520 to 1670 cm^−1^ and they can be attributed to the vibrational modes of the amide group present in OTA molecular structure [57]. Other bands due to OTA molecules are seen at 1310 cm^−1^ (stretching of aromatic rings), 1130 cm^−1^ and 1160 cm^−1^ (C-N ring stretching). Further evidence that aptamers captured OTA molecules can be found in the modification of the relative intensity of some vibrational modes of the aptamer, such as the stronger intensity of the ring stretching of guanine at 1395 cm^−1^ with respect the band at 1450 cm^−1^, or the intensity of the peak at 1424 cm^−1^ due to the H-bond deformation of Deoxyribosyl (C5’), which indeed increases with OTA concentration [57]. We have finally performed a control experiment in which only NRs and OTA at its highest concentration (10 µM) are mixed. After an incubation time of 2 h, the mixture is pipetted in a glass microcell and put under the Raman spectrometer. No NR aggregation is observed upon irradiation at 638 nm, even at full power (18 mW). Some sparse aggregates, spontaneously created, can be noticed at the bottom of the cell. The Raman signal recorded at full power on top of this aggregates is constant with time and, after normalization to power and integration time, is compared with the SERS signal in Figure 11b (brown line). We find an intensity difference of two orders of magnitude. Some low intensity bands are present between 1270 and 1330 cm^−1^, in the range of stretching vibrations due to aromatic rings, and from 1520 to 1670 cm^−1^ (vibrational modes of the amide group present in OTA molecular structure).

## 4. Conclusions

In the LIQUISOR methodology, optical forces are exploited to push metal nanoparticles dispersed in (bio)molecules solutions to induce a strong SERS emission from molecules embedded in the aggregates thus formed. This strategy allows high sensitivity molecular detection in liquid environment. In this work, we have extended the spectrum of applications of the LIQUISOR to detect hemeproteins (Hgb and Cat). Detection at physiological pH has been demonstrated, with sensitivities of few ng/mL (pM) for Hgb and of few µg/mL (10 nM) for Cat. Working on BSA, we have studied, with combined DLS, LSPR and SERS experiments, the growth kinetics of optically induced aggregates. We have found a monotonic increase of the SERS signal with the concentration, which paves the way for quantitative applications of the LIQUISOR methodology. A model has been developed that describes the process of nanorod/protein binding and size increase of the BIO-NRCs, supported by the experimental results. We have also shown that, in presence of BSA, gold nanorods can be optically printed on glass without any functionalization of the substrate, producing patterns that are stable also in absence of continuous irradiation. Finally, we have reported first results on the detection of Ochratoxin A taking advantage of nanorods functionalized with DNA aptamers. Aptamers have shown the ability to efficiently capture toxins in solutions and yield strong SERS when optical aggregation is obtained. These experiments represent a first step towards the addition of molecular specificity to the LIQUISOR methodology.

LIQUISOR is of rapid use (few minutes), experimentally simple (standard micro-spectrometers and commercial nanorods are used) and intrinsically scalable to lab-on-chip devices. Another advantage with respect to standard nanoantenna sensors, which are planar, is its 3D nature. Nanoparticles do aggregate in all three spatial dimensions inside the laser spot, allowing for a larger number of hot spots and of probed molecules with respect to 2D architectures. Several possible developments are envisaged for this technique such as: an investigation of the aggregation dynamics and of the LOD in Hgb, Cat and OTA; the use of more efficient nanostructures (e.g., core–shell, gold–silver alloys, and nanostars) [93,94,95,96] to improve the limit of detection; the exploration of new configurations for specific detection (e.g., employing functionalized surfaces to increase the affinity between BIO-NRCs and substrates); the integration in micro-fluidic chips; the use of laser beams in the optical transparency window of biological tissues that could enable the application of our scheme in combination with optical injection of nanoparticles into living cells for in vitro detection.

## Supplementary Materials

The following are available online at http://www.mdpi.com/1996-1944/11/3/440/s1, Figure S1: LSPR spectrum of the NRs; Figure S2: Plot of the optical forces acting on the NRs; Figure S3: Fits of the LSPR profiles of individual and aggregated NRs; Figure S4: SERS intensity at saturation of different characteristic Raman modes of BSA; Table S1: Best fit parameters (exponent) of the data in Figure S4. Supplementary Note 1, Hydrodynamic model of the aggregation process; Table S2: Position of the vibrational modes of Hgb measured in PB solution (0.1 mM), in powder state and through the LIQUISOR method (10 μM).
