# Supplementary material for: Optical Aggregation of Gold Nanoparticles for SERS Detection of Proteins and Toxins in Liquid Environment: Towards Ultrasensitive and Selective Detection
Source: Materials (Basel). 2018 Mar 17;11(3):440. doi: 10.3390/ma11030440 (PMC5873019; doi:10.3390/ma11030440)
Supplement: Supplementary file 1 [file materials-11-00440-s001.docx]

Supplementary Figure S1


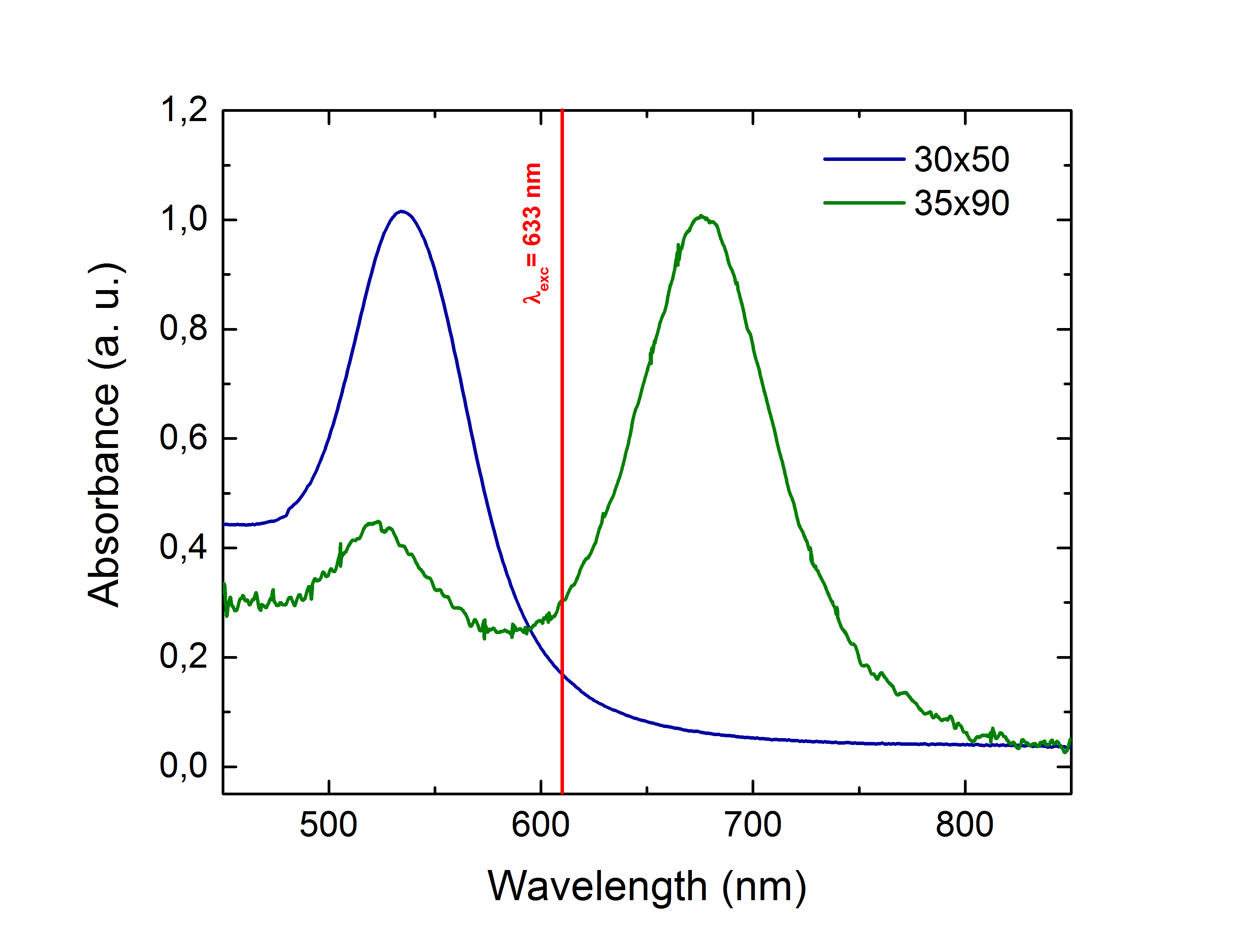


Figure S. 1 – Extinction spectra of gold NRs used for LIQUISOR experiments (green line, 35 nm diameter *×* 90 nm length – blue line 30 nm diameter $\times$ 50 nm length). Red line indicates the spectral position of the laser excitation. When the incident laser interacts with 35×90 NRs we are pre-resonant with respect to the LSPR peak and therefore the gradient force is repulsive ensuring a strong optical pushing effect [^[[1]](#endnote-1)^]. On the contrary when the laser interacts with 30×50 NR the gradient force is attractive but it is still not able to overcome the scattering force and therefore NRs are pushed along the laser beam direction also in this case. This is mostly due to the employment of low numerical aperture (~ 0.5) for our experiments.

Supplementary Figure S2
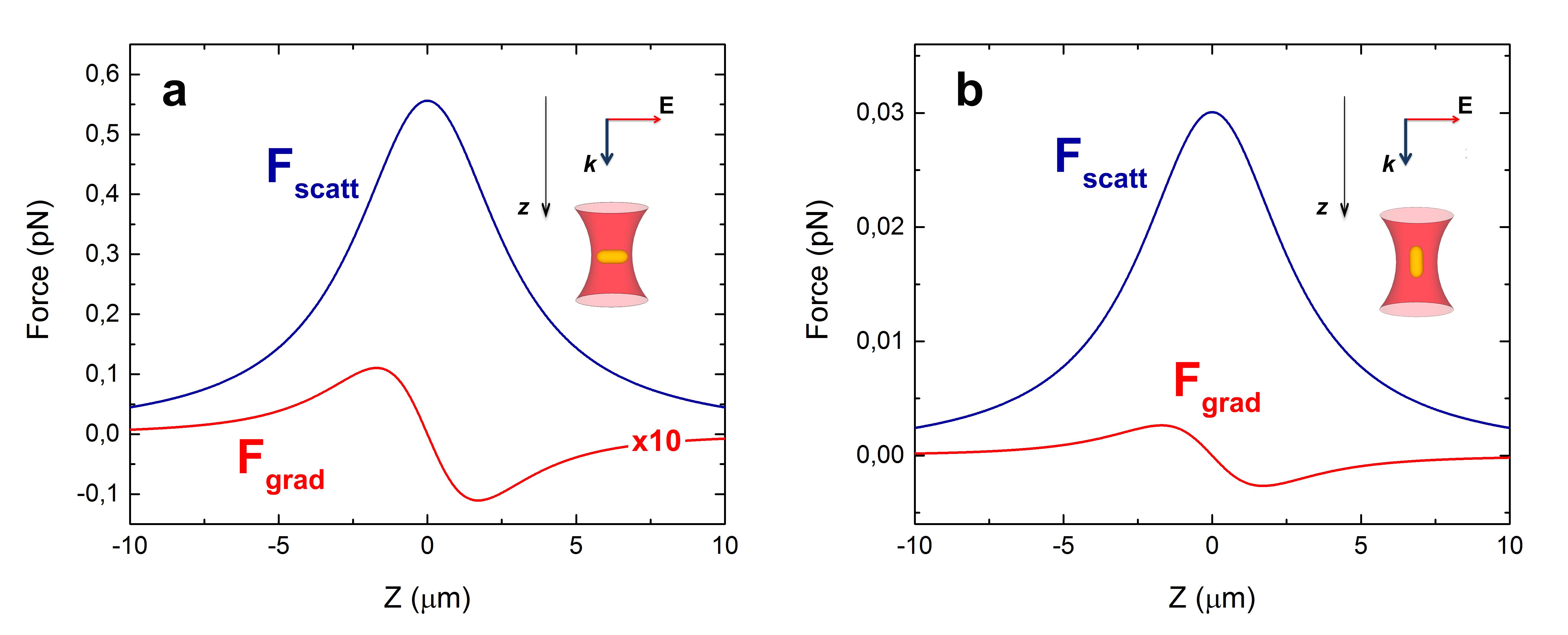


Figure S2: Radiation force ($F_{rad}$) acting on a gold nanorod is mainly composed by two principal contribution, a conservative one ($F_{grad}$) proportional to the gradient of the nanorod polarizability and a dissipative one ($F_{scatt}$) proportional to the extinction cross section [^[[2]](#endnote-2)^,^[[3]](#endnote-3)^]. Considering a laser beam emitting 638 nm (P = 13.5 mW) irradiating gold nanorods of 30 nm in diameter $\times$ 50 nm *via* a 50X long working distance objective (NA = 0.5), the dipole approximation can be considered valid. Details for the calculation of optical forces within this approximation can be found in ref [^[[4]](#endnote-4)^]. We considered our NRs as prolate spheroids immersed in water (refractive index *n_m_* = 1.33) and we use optical constants derived by Johnson and Christy [^[[5]](#endnote-5)^]. Calculations of optical forces are carried out for the two main configurations, i.e. when the field is polarized along the short (a) or the long axis (b) of the NRs. In both cases the gradient force (red lines) has an attractive nature. The scattering force (blue line) is, however, always more intense and dominates in the NRs-laser interaction, no matter how the nanorod is oriented. The predominance of the scattering force over the gradient one is at least one order of magnitude and it is insured exploiting a low numerical aperture objective. This keeps the intensity of gradient force low with respect to the scattering one, avoiding the optical trapping of the nanorods inside the laser focus region that eventually can occur when the laser wavelength is red shifted with respect to the LSPR maximum position. The net force balance acting on the rod is therefore always repulsive and leads to the pushing of the nanoparticles along the optical axis triggering the dynamic accumulation of the BIO-NRCs.

Supplementary Figure S3

Figure S. 3 – (a) Fits (continuous lines) of the experimental absorbance profiles (dots) of the NRs (30x50) diluted in water (black) and of the NRs mixed with BSA in PBS at 10^-4^ M (green). (b) Absorbance (black dots) and fit (continuous red line) of the NRs mixed with BSA in PBS at 5$\times$10^-8^ M. Measures carried out within the first 5 minutes from the mixing. The dotted lines highlight the position of the main peaks used for the fits.

Supplementary Figure S4


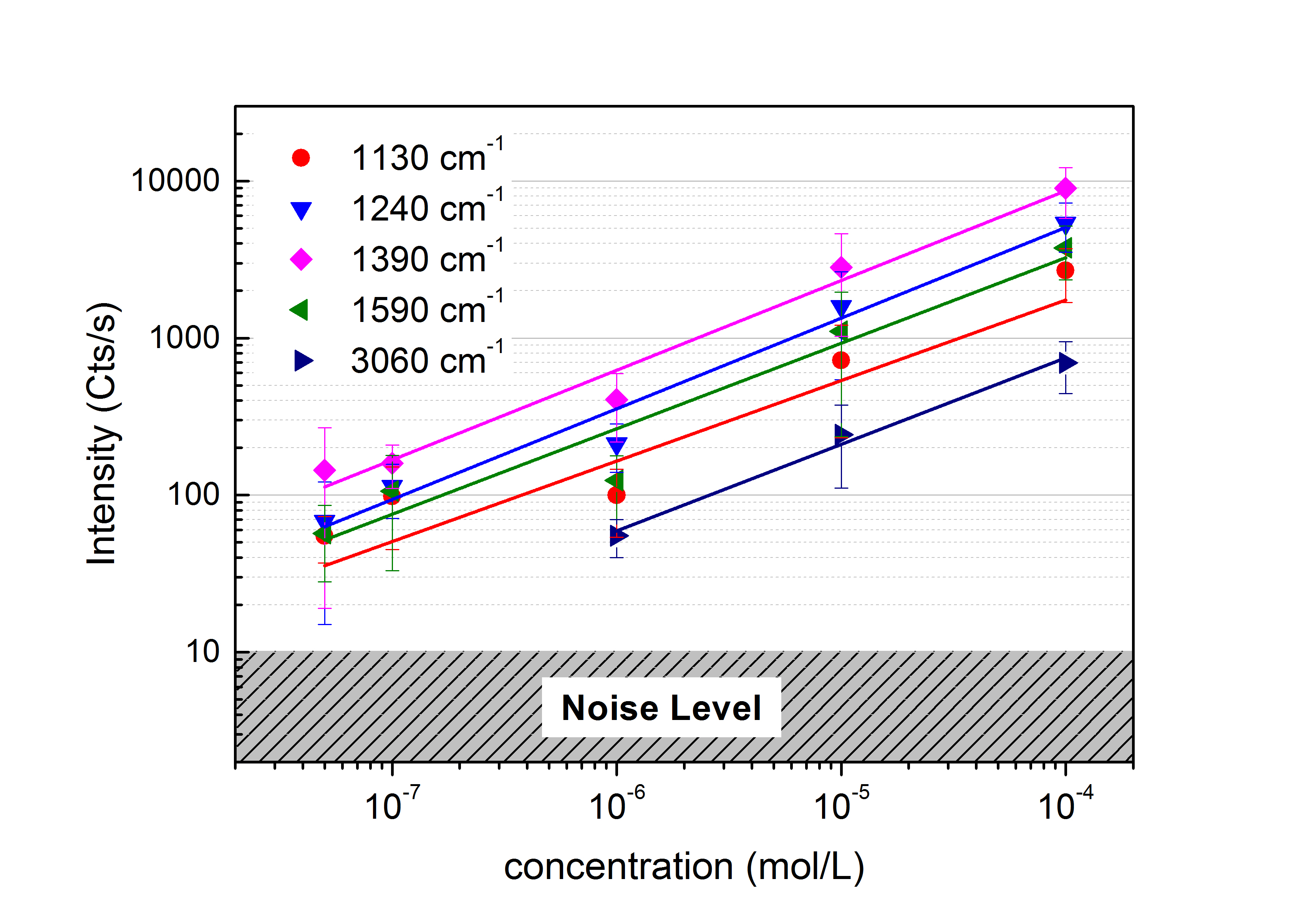


Figure S. 4 – SERS intensity at saturation for different characteristic Raman modes of BSA. Each point represents the averaged after background subtraction, from at least 4 saturated aggregates. Error bars are the standard deviation values calculated for each set of aggregates at the different concentrations. Solid lines are power law fits, whose exponents (reported in the table S1 below) are the same within the experimental fitting error to one found for Phe ring breathing mode (0.60 ± 0.04).

Supplementary Table 1

Table S1 – Best fit parameters (exponent) of the data in Figure S4.

| **Raman Mode** | **Mode assignment** | **Exponent** |
| --- | --- | --- |
| 1130 cm^-1^ | CN stretching [^[[6]](#endnote-6)^,^[[7]](#endnote-7)^] | 0,5 ± 0,1 |
| 1240 cm^-1^ | Amide III [6,7,^[[8]](#endnote-8)^] | 0,6 ± 0,1 |
| 1390 cm^-1^ | Aromatic amino acids COO^-^ stretching [6,7,^[[9]](#endnote-9)^] | 0,59 ± 0,05 |
| 1590 cm^-1^ | Phe [6,7] | 0,6 ± 0,1 |
| 3060 cm^-1^ | Aromatic CH stretching [^[[10]](#endnote-10)^] | 0,55 ± 0,05 |

Supplementary Note 1

*Hydrodynamic model of the aggregation process*

We assume that the rate of laser volume filling depends on the diffusion coefficient *D* of the BIO-NRCs which, in turn, is related to the probability of entering in the laser beam, and on the speed at which these objects can reach the bottom of the glass microcell. The value of this speed is the equilibrium velocity that we can retrieve from the balance of forces acting on the nanostructure: the radiation force ($F_{rad}$) and the gravity ($F_{g}$) that point downward, and the buoyancy ($F_{b}$) forces and the drag force ($F_{drag}$) that point upward. Considering the BIO-NRCs as nanospheres with radius equal to MHR, we can use the information retrieved from DLS measurements to model the trend of the saturation time as a function of the concentration. First of all we have to find the dependence of the filling rate ($v_{fill}$) on the MHR. The diffusion coefficient is given by the fluctuation-dissipation theorem [^[[11]](#endnote-11)^]:

|  | $D=\frac{k_{B}T}{\gamma},$ | (1) |
| --- | --- | --- |

where $\gamma=6\pi\eta R_{H}$ is the Stokes’ particle friction coefficient for a spherical particle of radius $R_{H}$. Therefore we have $D\propto R_{H}^{-1}$. On the other hand the force balance equation leads to:

|  | $F_{rad}+{(F}_{g}-F_{b})=F_{drag}.$ | (2) |
| --- | --- | --- |

Using the Stokes law for the calculation of the drag force on a nanospheres with radius $R_{H}$, we have:

|  | $F_{drag}=\gamma v_{eq}=6\pi\eta R_{H}v_{eq}.$ | (3) |
| --- | --- | --- |

Considering that $F_{rad}$ is directly proportional to the nanoparticle polarizability $\alpha$, which is proportional to the volume $V\propto R_{H}^{3}$, we have that $F_{rad}\propto R_{H}^{3}$, while for ${(F}_{g}-F_{b})$ is given by:

|  | ${(F}_{g}-F_{b})=(\rho_{p}-\rho_{m})gV\propto R_{H}^{3},$ | (4) |
| --- | --- | --- |

where $\rho_{p}$ and $\rho_{m}$ are the density of the particle and of the medium respectively. Therefore we can say that the left part in Eq. 6 is proportional to the cube of $R_{H}$ and combining the result with Eq. 3, we can assert:

|  | $v_{eq}\propto R_{H}^{2},$ | (5) |
| --- | --- | --- |

and finally we have that the rate of nanostructures filling the laser volume is related to the product of the diffusion coefficient and the equilibrium velocity:

|  | $v_{fill}\propto Dv_{eq}\propto R_{H}.$ | (6) |
| --- | --- | --- |

The average experimental $v_{fill}$ can be estimated by the ratio between the number of BIO-NRCs ($N_{Nano}$) necessary to fill the diffraction limited volume probed by the microscope objective ($V_{laser}$) and the measured saturation time ($T_{sat}$). $V_{laser}$ can be estimated calculating the volume of Point Spread Function (PSF) [^[[12]](#endnote-12)^] of a TEM_00_ laser beam at wavelength λ, focused in air by an objective with numerical aperture *NA*. The PSF is well approximated by a prolate ellipsoid having semi-axes *b_1_* = *b_2_* = 0.61 × λ/*NA* and *b_3_* = 2 × *λ/NA*^2^, where *b_3_* is the semi-axis in the light propagation direction. The volume of the focused laser spot, therefore, will be $V_{laser}$ = (4π/3)*b_1_b_2_b_3_* ∼ 2λ^3^/*NA*^4^. For λ = 633 nm and *NA*= 0.9, we find $V_{laser}$~ 1.2 μm^3^. On the other hand, $N_{Nano}$ can be estimated in the ideal situation in which we consider a saturated aggregate where the NRs, surrounded by a protein bilayer, are closely packed and totally fill the focal laser spot. Thus $N_{Nano}$ is given by the ratio between $V_{laser}$ and the volume of one BIO-NRC, whose volume is approximated to $V_{BIO-NRC}={4\pi}/3R_{H}^{3}$.

Mixing NRs with BSA 0.1 mM leads to stable BIO-NRCs with $R_{H}$ equal to 65 nm and the observed averaged saturation time for SERS intensity is $T_{sat}^{0.1mM}$ = 3200 ± 660 s. Using these values we can estimate the number of nanostructures in the laser spot after saturation ($N_{Nano}^{0.1mM}\sim{10}^{3}$) and rate of filling $v_{fill}\sim$ 0.33 ± 0.07 nanostructures/s. Exploiting Eq. 3 we can rescale this value for lower concentrations taking into account the trends for $R_{H}$ extrapolated from DLS measurements reported in Figure 3 of the main text. Considering the operational time for LIQUISOR starting from 120s (the time necessary to prepare the sample and put it under the spectrometer), we can calculate the saturation time at 10 µM, 1 µM, 100 nM and 50 nM with the rescaled $v_{fill}$ for each concentration. Values obtained from the model for the laser spot filling (red triangles in Figure 5 of main text) are quite in agreement with the observed values (black dots in Errore. L'origine riferimento non è stata trovata.). The discrepancy between experimental and calculated values has to be ascribed to a combination of different issues: (i) the spherical approximation for the BIO-NRCs shape; (ii) the laser-induced heating of the NRs, which fosters the nanoparticles gathering and the protein uptake, this enlarging the dimensions of BIO-NRCs [4]; (iii) the dependence of the diffusion coefficient on the density n of diffusing BIO-NRCs. Indeed even if the initial concentration of NR mixed to protein solutions is kept constant, we saw the different dynamics of BIO-NRCs at different concentration leads to nanostructures of increasing dimensions as the concentration is lowered and as a result the density of diffusing objects decrease. This means that the model described above overestimates $v_{fill}$ providing a shorter saturation time for SERS intensity with respect to the actual one, that is what we observe in igure 5 of the main text.

Supplementary Table 2

Table S2 -Vibrational modes (cm^-1^) of Hgb measured in PBS solution (0.1 mM), in powder state and through the LIQUISOR method (10 μM). Modes assignment is carried out following the literature.

| **Raman of Hgb in PBS (0.1 mM)** | **Raman of Hgb in powder state** | **SERS of Hgb in PBS (10 µM)** | **Tentative mode assignment of the Raman modes** |
| --- | --- | --- | --- |

| 664 | 663 | 668 | δ(pyr deform)_sym_, [^[[13]](#endnote-13)^,^[[14]](#endnote-14)^] |
| --- | --- | --- | --- |
| 721 | 723 | 721 | δ(COO^-^) [12] |
| 750 | 750 | 750 | ν(pyr breathing), ν_15_ [12,13] |
| 784 | 790 | 786 | Trp:indole sym. breathe + ν(pyr breathe), ν_6_ [12] |
| 924 | -- | 924 | C-COO^-^ stretch. [12] |
| -- | -- | 967 | C-C stretch. [12] |
| 997 | 999 | 994 | Phe [12] |
| 1080 | -- | 1079 | =C_2vynil_H + Trp [4,12] |
| 1125 | 1123 | 1125 | C-N and C-C stretch [4,12] |
| 1170 | 1173 | 1162 | ν(pyr half-ring)_asym._, ν_30_ +Tyr+Phe [4,12, 13] |
| 1214 | -- | 1214 | δ(CH) [12] |
| 1235 | -- | 1235 | Trp + Amide III [4,12] |
| 1254 | 1248 | 1263 | Amide III [4] |
| 1306 | 1309 | 1309 | CH_2_ wag [4,12] |
| 1340 | 1340 | -- | CH_3_ def. CH2 twist. CH bending [4,12] |
| -- | 1366 | 1363 | Tyr [4] |
| 1393 | 1395 | 1388 | ν (COO^-^) [4] |
| -- | 1445 | 1442 | δ(CH_2_/CH_3_) [4,12] |
| -- | -- | 1486 | Trp + Phe [4,12] |
| 1543 | 1546 | 1543 | Amide II + Trp [4,12,^[[15]](#endnote-15)^] |
| -- | -- | 1564 | Trp[4,12] |
| 1582 | 1579 | 1584 | Phe + Tyr [12] |
| 1610 | 1603 | 1608 | Phe + Tyr [12] |
| -- | 1623 | 1617 | Pyr ν (C=C) [12] |
| 1655 | -- | 1655 | Amide I [4,14] |

References

1. Arias-González, J.R.; Nieto-Vesperinas M. Optical forces on small particles: attractive and repulsive nature and plasmon-resonance conditions. *J. Opt. Soc. Am. A,* **2003**, 20, 1201–1209, DOI: https://doi.org/10.1364/JOSAA.20.001201. [↑](#endnote-ref-1)
2. Maragò, O.M.; Jones, P.H.; Gucciardi, P.G.; Volpe, G.; Ferrari, A.C. Optical trapping and manipulation of nanostructures. *Nat. Nanotechnol.* **2013**, 8, 807–819, DOI: 10.1038/nnano.2013.208. [↑](#endnote-ref-2)
3. Xu, H.; Käll, M. Surface-plasmon-enhanced optical forces in silver nanoaggregates. *Phys. Rev. Lett.* **2002**, 89, 246802, DOI: https://doi.org/10.1103/PhysRevLett.89.246802. [↑](#endnote-ref-3)
4. Fazio, B.; D’Andrea, C.; Foti, A.; Messina, E.; Irrera, A.; Donato, M.G.; Villari, V.; Micali, N.; Maragò, O.M.; Gucciardi, P.G. SERS detection of Biomolecules at Physiological pH via aggregation of Gold Nanorods mediated by Optical Forces and Plasmonic Heating, *Sci. Rep. UK* **2016**, 6, 26952, DOI: 10.1038/srep26952. [↑](#endnote-ref-4)
5. Johnson, P.B.; Christy, R.W. Optical constants of the noble metals. *Phys. Rev. B* **1972**, 6, 4370–4379, DOI: https://doi.org/10.1103/PhysRevB.6.4370. [↑](#endnote-ref-5)
6. Chen, M.C.; Lord, R.C. Laser-Excited Raman Spectroscopy of Biomolecules. VIII. Conformational Study of Bovine Serum Albumin. *J. Am. Chem. Soc.* **1976**, 98, 990-992. [↑](#endnote-ref-6)
7. Lin, V.J.C.; Koenig, J.L. Raman Studies of Bovine Serum Albumin. *Biopolymers* **1976**, 15, 203-218, DOI: 10.1002/bip.1976.360150114. [↑](#endnote-ref-7)
8. Cavalu, S.; Cinta-Pinzaru, S.; Leopold, N.; Kiefer, W. Raman and Surface Enhanced Raman Spectroscopy of 2,2,5,5-Tetramethyl-3-pyrrolin-1-yloxy-3-carboxamide Labeled Proteins: Bovine Serum Albumin and Cytochrome C. *Biopolymers* **2001**, 62, 341, DOI: 10.1002/bip.10002. [↑](#endnote-ref-8)
9. Navarra, G.; Tinti, A.; Leone, M.; Militello, V.; Torreggiani, A. Influence of metal ions on thermal aggregation of bovine serum albumin: Aggregation kinetics and structural changes. *J. Inorg. Biochem.* **2009**, 103, 1729–1738, DOI: https://doi.org/10.1016/j.jinorgbio.2009.09.023. [↑](#endnote-ref-9)
10. Socrates, G. *Infrared and Raman Characteristic Group Frequencies: Tables and Charts*, 3rd Edition. John Wiley & Sons, 2004, ISBN: 978-0-470-09307-8. [↑](#endnote-ref-10)
11. Jones, P.; Maragó O.M.; Volpe G., Optical tweezers: Principles and applications. Cambridge University Press, 2015, ISBN: 9781107279711. [↑](#endnote-ref-11)
12. Novotny, L.; Hecht B. Principles of Nano-optics, 2nd Ed., Cambridge University Press, Cambridge, 2012, ISBN: 978-1-107-00546-4. [↑](#endnote-ref-12)
13. Drescher, D.; Büchner, T.; McNaughton, D.; Kneipp, J. SERS reveals the specific interaction of silver and gold nanoparticles with hemoglobin and red blood cell components. *Phys. Chem. Chem. Phys.* **2013**, 15, 5364, DOI: 10.1039/C3CP43883J. [↑](#endnote-ref-13)
14. Hu S., Smith K. M. and Spiro T.G., Assignment of protoheme resonance Raman spectrum by heme labeling in myoglobin. *J. Am. Chem. Soc.* **1996**, 118, 12638-12648, DOI: 10.1021/ja962239e. [↑](#endnote-ref-14)
15. Pelton, J.T.; McLean, L.R. Spectroscopic Methods for Analysis of Protein Secondary Structure. *Anal. Biochem.* **2000**, 277, 167–176, DOI: https://doi.org/10.1006/abio.1999.4320. [↑](#endnote-ref-15)
